# Green Synthesis of Silver Nanoparticles Using *Hypericum perforatum* L. Aqueous Extract with the Evaluation of Its Antibacterial Activity against Clinical and Food Pathogens

**DOI:** 10.3390/pharmaceutics14051104

**Published:** 2022-05-21

**Authors:** Abdalrahim Alahmad, Wael A. Al-Zereini, Tahani J. Hijazin, Osama Y. Al-Madanat, Ibrahim Alghoraibi, Omar Al-Qaralleh, Samer Al-Qaraleh, Armin Feldhoff, Johanna-Gabriela Walter, Thomas Scheper

**Affiliations:** 1 Institut für Technische Chemie, Leibniz Universität Hannove, Callinstraße 5, 30167 Hannover, Germany; walter@iftc.uni-hannover.de (J.-G.W.); scheper@iftc.uni-hannover.de (T.S.); 2 Department of Biological Sciences, Faculty of Scince, Mutah University, P.O. Box 7, Mutah 61710, Jordan; hijazin@mutah.edu.jo (T.J.H.); omarqaralleh.1995@gmail.com (O.A.-Q.); 3 Department of Chemistry, Faculty of Scince, Mutah University, P.O. Box 7, Mutah 61710, Jordan; 4 Physics Department, Faculty of Science, Damascus University, Damascus P.O. Box 30621, Syria; ibrahim.alghoraibi@gmail.com; 5 Faculty of Medicine, Mutah University, P.O. Box 7, Mutah 61710, Jordan; 75garalleh@gmail.com; 6 Institut für Physikalische Chemie und Elektrochemie, Leibniz Universität Hannove, Callinstraße 3A, 30167 Hannover, Germany; armin.feldhoff@pci.uni-hannover.de

**Keywords:** silver nanoparticles, green synthesis, *Hypericum perforatum* L., antibacterial

## Abstract

The rapid development of nanotechnology and its applications in medicine has provided the perfect solution against a wide range of different microbes, especially antibiotic-resistant ones. In this study, a one-step approach was used in preparing silver nanoparticles (AgNPs) by mixing silver nitrate with hot *Hypericum perforatum* (St. John’s wort) aqueous extract under high stirring to prevent agglomeration. The formation of silver nanoparticles was monitored by continuous measurement of the surface plasma resonance spectra (UV-VIS). The effect of St. John’s wort aqueous extract on the formation of silver nanoparticles was evaluated and fully characterized by using different physicochemical techniques. The obtained silver nanoparticles were spherical, monodisperse, face-centered cubic (fcc) crystal structures, and the size ranges between 20 to 40 nm. They were covered with a capping layer of organic compounds considered as a nano dimension protective layer that prevents agglomeration and sedimentation. AgNPs revealed antibacterial activity against both tested Gram-positive and Gram-negative bacterial strains causing the formation of 13–32 mm inhibition zones with MIC 6.25–12.5 µg/mL; *Escherichia coli* strains were resistant to tested AgNPs. The specific growth rate of *S. aureus* was significantly reduced due to tested AgNPs at concentrations ≥½ MIC. AgNPs did not affect wound migration in fibroblast cell lines compared to control. Our results highlighted the potential use of AgNPs capped with plant extracts in the pharmaceutical and food industries to control bacterial pathogens’ growth; however, further studies are required to confirm their wound healing capability and their health impact must be critically evaluated.

## 1. Introduction

Recently, there has been an increase in the number of emerged antibiotic-resistant bacterial strains, the number of multidrug-resistant tumor cells, and the number of oxidative stress-associated disorders; an increase that elevates the burden of treating such ailments. Moreover, the usage of some synthetic drugs is associated with adverse effects on human health [1]. The introduction of bio- and nanotechnology-based techniques in medical research led to nanomaterials’ application in treating, diagnosis, control, and modulation of biological systems [2]. Intriguingly, nanoparticles were synthesized through chemical and physical methods; they are expensive, environmentally un-favorable, and toxic, hindering the compatibility of resulting NPs for biological applications [3]. Using biosystems such as microorganisms and plants as manufacturers of nanomaterials is considered a more safe and more eco-friendly method [4]. However, using plant extracts is more expedient than using microorganisms to overcome the issues of media sterilization, cultivation, and maintaining microbial cells [5]. Plant extracts provide agents that act as reductants and stabilizers during the synthesis of nanoparticles; phenols, alkaloids, tannins, flavonoids, and saponins among others are examples of such reagents [6]. The leave extracts of *Aloe vera* [7], *Capsicum annum* [8], *Euphorbia hirta* [9], *Acalypha indica* [10], *Garcinia mangostana* [11], *Clerodendrum inerme* [12], *Curcuma longa* [13], *Capparis zeylanica* [14], and *Calendula officinalis* [15], the fruit extract of *Carica papaya* [16], the latex and seed extract of *Jatropha curcas* [17], and bark extract of *Cinnamon zeylanicum* [18], among other plants, were used to photosynthesize silver nanoparticles (AgNPs) in a size range of 25–100 nm with antioxidant, cytotoxic, and anti-Gram-positive and -Gram-negative bacterial activities.

The ability of phyto-synthesized AgNPs to exhibit a broad-spectrum antibacterial activity and to overcome the antibiotic resistance issue in microorganisms might be attributed to their intrinsic therapeutic characteristics with a multi-target effect [19,20]. In comparison to gold, platinum, iron, and zinc nanoelements, AgNPs exhibit very high bioactivity, low levels of cytotoxicity when used individually or when been combined with various antibiotics to overcome the resistance of bacteria to many drugs [21]; they alter microbial cell membrane and wall structures [22,23], induce reactive oxygen species (ROS) production, DNA damage, and lead to cellular apoptosis and necrosis [24,25,26]. They were applied in pathogen detection and diagnosis, topical wound healing, drug delivery, the coating of materials and devices including textile, food, and medical instruments and applications [27,28]. Due to their antibacterial activity, they are used in cardiovascular and dental implants [29].

*Hypericum perforatum* L. (St. John’s wort) has been used as a remedy to treat the digestive tract, skin wounds, burns, cancer, AIDS, and psychological diseases [30,31,32,33,34,35]. It contains compounds with potential antimicrobial, antiviral, antioxidant, antidiabetic, anti-inflammatory, analgesic, and anticancer activities [36]; activities that were attributed to their content of hypericin, pseudohypericin, hyperforin, bioflavonoids, and flavonoids [37,38,39,40,41]. There are contradictory reports on its antibacterial activity; some reports documented its broad antibacterial activity [40,41] while others demonstrated that *H. perforatum* extract was ineffective in inhibiting bacterial growth [42].

Several studies highlighted the role of *H. perforatum* aqueous extract in the reduction of metal ions to nanoelements and in stabilizing the biogenic particles. Its extract was used in reducing gold (III) chloride hydrate (HAuCl_4_) and silver nitrate (AgNO_3_) to AuNPs with antidepressant and antioxidant activity [43,44], and AgNPs with antibacterial, antioxidant, and anticancer activities [4,41,42,45]. Therefore, the present study aimed to investigate the role of AgNPs capped with *H. perforatum*’s metabolites in controlling bacterial growth and wound healing.

In the current study, the aqueous extract of *H. perforatum* was used to reduce AgNO_3_ into AgNPs. The synthesis procedure of the silver nanoparticles was performed based on our previous report [4], with some modifications for the temperature and concentrations of plant extracts as well as silver precursor aiming to produce small AgNPs with a large surface-to-volume ratio. Compared to our previous procedure, the concentration of the silver nitrate was increased to 0.5 M and was added to a known concentration (~2000 ppm) of plant extract (previously the concentration of the plant extract was not known as it was used directly after decoction). The reaction temperature was increased and maintained at 80 °C for 4 h. The rate of silver nitrate addition was reduced to around 50 μl/min for around 3.5 h with the increasing of the stirring rate to 750 rpm. The preparation was left for a further 30 min under stirring until completion of the reaction. By modifying all these conditions, we observed a small variation in the particle size (20–40 nm), while previously its range was between 20–60 nm. The coating of the nanoparticles and their stabilization by the plant phytochemical constituents was confirmed via chemical characterization and their antibacterial efficiency against clinical and food pathogenic isolates was evaluated and compared with that of the plant extract; their effect on the growth kinetics and killing time of tested *Staphylococcus aureus* was elaborated. Herein, the effect of *H. perforatum*-coated AgNPs on wound healing was investigated for the first time.

## 2. Materials and Methods

### 2.1. Chemicals and Instrumentation

Aerial parts of *H. perforatum* L. were collected in July–August from the Ghab Plain in Syria and harvested during the flowering season. The sample was identified and authenticated by Dr. Watfeh of the Faculty of Agriculture at Damascus University. Silver nitrate was purchased from Sigma Aldrich. Whatman filter paper (90 mm) was purchased from GE Healthcare Life Sciences (Freiburg Germany). Deionized water (18.2 MΩ cm, 25 °C) was obtained from a Millipore Mill-Q system, Sartorius (Goettingen, Germany).

The absorption spectra of AgNPs, immediately after synthesis, after one month, and after 8 months of storage, were recorded using a NanoDrop Spectrophotometer (ND1000 from PeQLab). Dynamic light scattering and Zeta potential using Bettersize S3 plus from Anton Paar was performed. This was followed by Fourier transform infrared spectrophotometry using a Bruker FT-IR Vertex 80 v spectrometer. X-ray diffraction (XRD) wase done using a Bruker D8 Advance diffractometer. A thermal gravimetric analysis (TGA) was performed using a TGA/DSC 3+ from Mettler-Toledo, from 25 to 1000 °C at a rate of 1 °C per minute with N_2_ gas flow. Scanning electron microscopy (SEM) and energy-dispersive X-ray spectroscopy (EDXS) were done using a JEOL JSM-6700F. Transmission electron microscopy (TEM) was performed with the use of a JEOL JEM-2100F-UHR, at an acceleration voltage of 200 kV. The surface morphology was determined by the atomic force microscopy measurements (AFM, Nanosurf easyScan2, Liestal, Switzerland). The AFM measurements were performed in contact mode.

NTA measurements were performed with a NanoSight LM10 (NanoSight, Amesbury, UK) equipped with a sample chamber with a 635 nm laser and a Viton fluoroelastomer O-ring. The samples were injected into the sample chamber with sterile syringes (Carl Roth GmbH, Karlsruhe, Germany) until the liquid reached the tip of the nozzle. All measurements were performed at room temperature. The software used for capturing and analyzing the data was the NTA 2.0 Build 0033. The samples were measured for 60 s with manual shutter and gain adjustments. The “single shutter and gain mode” was used to capture the monodisperse AgNPs.

### 2.2. Synthesis of Hypericum Perforatum L. aqueous Extract Mediated Silver Nanoparticles (AgNPs) and Their Chemical Characterization

The synthesis of AgNPs was done as mentioned previously [4], with some modifications. Briefly, *H. perforatum* aerial parts were cleaned and ground to a fine powder. 3 g of the pulverized sample were boiled in 700 mL distilled water for 3 h, and the supernatant was filtered using a 0.22 μm membrane filter. The resulting yellow-brown extract was freeze-dried. Preparation of AgNPs was achieved by adding 10 mL of AgNO_3_ (0.5 M) dropwise to plant extract solution (0.075 g of the plant extract in 40 mL deionized water) with stirring at 750 rpm and 80 °C. The formation of AgNPs was confirmed by the change of preparation color from pale yellow to dark brown, and the preparation was left in a dark place while cooling. The resulting nanoparticles were isolated, washed with deionized water, and centrifugated at 15,000 rpm for one hour, and the process was repeated at least four times. The purified AgNPs were chemically characterized using ultraviolet light spectroscopy (UV-VIS), dynamic light scattering (DLS) and zeta potential, Fourier transform infrared spectroscopy (FTIR), X-ray diffraction (XRD), scanning electron microscopy (SEM), transmission electron microscopy (TEM), energy disperse X-ray spectroscopy (EDX), atomic force microscopy (AFM) and nanoparticle tracking analysis (NTA).

The phytochemical constituents of *H. perforatum* extract, which might have a role in reducing AgNO_3_ and stabilizing the formed AgNPs, were determined in a previous work by high-performance liquid chromatography equipped with a diode array detector (HPLC-DAD) coupled with UV-visible spectrophotometry at a full spectrum (200–800 nm) [46]. Detection was carried out at 260 nm for phloroglucinols, 590 nm for naphthodianthrones, and 350 nm for other flavonols, flavones, and chlorogenic acids.

### 2.3. Antibacterial Activity

The green synthesized AgNPs were tested for their antibacterial activity by a well diffusion test, and the lowest concentration needed to inhibit bacterial growth (MIC) was determined by a micro-broth dilution assay; both assays were performed as described in Al-Zereini [47] and according to Clinical and Laboratory Standards Institute guidelines, with some modifications. AgNPs were tested against the Gram-negative bacteria [*Pseudomonas aeruginosa* (ATCC 13048), β-lactamase *Klebsiella pneumoniae* (clinical isolate), extended-spectrum β-lactamase *Escherichia coli* (ESBL, clinical isolate), and *Escherichia coli* (ATCC 25922)] and the Gram-positive bacteria [*Staphylococcus aureus* (ATCC 43300), *Bacillus cereus* (ATCC 11778) and *B. subtilis* (ATCC 6633)]. The clinical isolates were supplied by Dr. Haitham Qaralleh of the Department of Medical Laboratory Sciences at Mutah University and were identified by a BIOMÉRIEUX VITEK^®^ 2 system. Overnight, the bacterial culture was seeded in Muller Hinton agar plates (MHA) (Oxoid, Hampshire, UK) at a cell density of 10^6^ cells/mL and in which 6 mm diameter wells were made using a sterile Cork borer. To each well in the bacterial agar plates, either 120 μL aliquots (100 μg/well), 60 μL aliquots (50 μg/well) from AgNPs preparation, or 100 μL aqueous plant extract (2.5 mg/well) were applied. As a positive control, streptomycin (10 μg/disc, Bio Basic Inc., Markham, ON, Canada) was used and the results are presented as means of triplicate tests ± SD.

However, in the micro-broth dilution assay, AgNPs and the positive control were tested starting from 100 μg/mL and 50 μg/mL, respectively. Samples were serially diluted in 100 µL Muller Hinton broth (MHB), to which 100 μL of the tested bacterial strain was added to each well at a cell density of 2 × 10^6^ cells/mL. The preparation was incubated for 24 h at 37 °C. Aliquots from the preparations in micro-broth dilution assay, where there were no visible growths, were platted on agar plates with proper medium and incubation for 24–48 h to determine if the nanoparticle has a bactericidal or bacteriostatic effect.

### 2.4. Effect of AgNPs on Growth Kinetics of S. aureus

Based on the AgNPs antibacterial activity results, *S. aureus* was selected to study the effect of nanoparticles on the bacterial growth kinetics. *S. aureus* was cultivated at a cell density of 10^6^ cells/mL in sterile test tubes containing 10 mL of MHB (Oxoid, UK). Different concentrations of AgNPs (1, 3, 6, 12, and 24 μg/mL) were added to bacterial culture test tubes with incubation for 24 h on an orbital shaker (Forma-Thermo electron cooperation, Waltham, MA, USA) at 37 °C and 150 rpm. A bacterial culture test tube without AgNPs was used as a negative control. During the cultivation period, 1 mL sample was withdrawn after 3 h intervals and up to 24 h to measure bacterial growth in terms of increase in the optical cell density at λ_600nm_ (OD_600nm_) using a UV/Vis spectrophotometer (Novaspec Model. 80-2088-64, Pharmacia Biotech, UK). The growth curve was established by plotting OD_600nm_ values against the treatment time. The specific growth rate was calculated according to the formula:

µ = (ln(X_2_) − ln(X_1_))/(t_2_ − t_1_)

where µ is the specific growth rate, X_1_ and X_2_ are the OD_600nm_ at time t_1_ and t_2_ of the bacterial culture. The experiment was performed in triplicate and the results are presented as means of three independent tests ± SD.

### 2.5. Estimation of Antibacterial Activity of AgNPs in Terms of CFU

Different concentrations of AgNPs (1, 3, 6, 12, and 24 µg/mL) were used to evaluate their effect against *S. aureus* growth in terms of colony-forming units (CFU) on Muller Hinton agar plates. The bacterium was grown overnight and then diluted to a cell density equivalent to 0.5 McFarland (≈1.5 × 10^8^ CFU/mL). They were diluted to 10^5^ fold and 100 μL were spread on solid agar plates containing different AgNPs concentrations. The plates were incubated for 24 h at 37 °C and the grown colonies were photographed.

### 2.6. Time-Kill Kinetics Assay

The time-kill assay was performed for the *S. aureus* culture exposed to ½ MIC, MIC, and 2 MIC concentrations of AgNPs following the CLSI guideline [48]. An overnight bacterial culture was centrifuged at 4000 rpm for 1 h to harvest the cells (Combi-514R, Hanil Scientific Inc., Gimpo, Korea). The harvested cells were suspended in sterile normal saline (0.085% NaCl, w/v) and adjusted to 0.5 McFarland (≈1.5 × 10^8^ CFU/mL). Aliquots of 1 mL from the prepared suspension were inoculated into test tubes containing 10 mL MHB (Oxoid, UK), with final concentrations of the AgNPs equal to ½ MIC, MIC, and 2 MIC. All tubes were incubated at 37 °C and samples were taken at intervals of 30 min starting at the time of inoculation and up to 300 min. The estimation of the number of surviving bacteria was carried out by spreading 100 μL from appropriate bacterial dilutions in sterile normal saline onto the top of MHA plates with incubation at 37 °C for 24 h; the number of bacterial colonies was counted and expressed as CFU/mL. Time-kill curves were constructed by plotting the Log_10_ (CFU/mL) against the exposure time (minutes). The assay was performed in triplicate and the results are presented as means of three independent tests ± SD.

### 2.7. Wound Migration Assay

A wound migration assay was performed using the normal human dermal fibroblast cell line (ATCC^®^ PCS-201-012) and as described in Wadhwani et al. [49]. Cells were cultured in a 12-well microtiter plate as a monolayer, and they were seeded at a cell density of 5 × 10^4^ cells/mL. The cells were grown for 24 h in Dulbecco’s Modified Eagle Medium (DMEM; Gibco, Mannheim, Germany) supplemented with 10% heat-inactivated fetal calf serum (Gibco, Germany), L-glutamine, 65 μg/mL of penicillin G, and 100 μg/mL of streptomycin sulfate. They were incubated at 37 °C in a humidified atmosphere containing 5.0% CO_2_. A scratch of a uniform size was made in each well using a sterile micro-tip; the culture medium was aspirated from each well, the well was washed with a sterile phosphate buffer solution (pH 7.4), and fresh media with different concentrations of AgNPs (1, 3, 5, and 10 µg/mL) were added to each well seeded with the cell line. Two wells containing a fresh medium without the test compounds were used as untreated controls. The culture was photographed at 0 time and after 24 h using an inverted microscope (Nikon, Shinjuku-ku, Japan). Wound migration (closure) was measured by the following equation:

% Wound migration = 1 − (area of the wound at T_S_/area of the wound at T_0_) × 100

where T_S_ is 24 h from wounding and T_0_ is 0 h wounding.

## 3. Result and Discussion

### 3.1. Chemical Characterization of Hypericum perforatum L. Phytochemicals-Capped AgNPs

**Figure 1:**
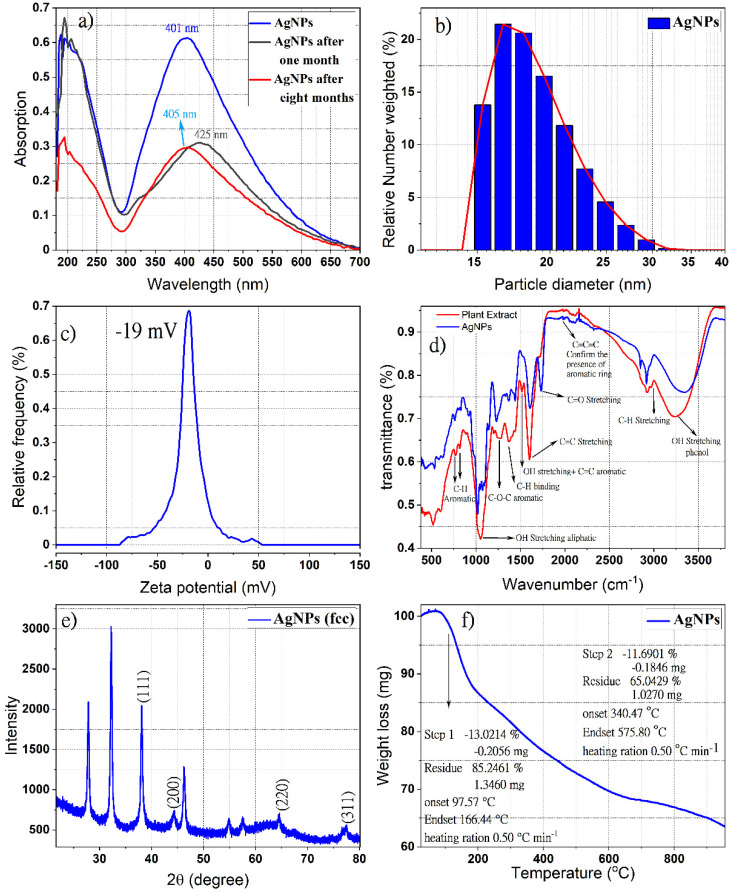
UV−Vis spectrum of ingredients-stabilized silver nanoparticles (AgNPs) using *Hypericum perforatum* L. Aqueous Extract (**a**); hydrodynamic diameter distribution curve (**b**); zeta potential distribution curve (**c**); ATR-IR spectra of the resulted AgNPs and *H. perforatum* L. aqueous Extract (**d**); X-ray diffraction pattern of resulting AgNPs colloidal (**e**); thermal gravimetric analysis of the resulting phytochemicals-AgNPs (**f**).

The aqueous extract is used as a reducing agent for silver ions into metallic silver, as well as a stabilizer to protect the silver nanoparticles (AgNPs) and prevent their aggregating. During the preparation process, the formation of the metallic silver particles via the silver ions (Ag^+^) reduction with the St John’s wort aqueous extract was observed by the color change to a light or dark brown. Figure 1a shows the UV-Vis spectra of phyto-capped AgNPs at various times (fresh, one month, and eight months after preparations). The absorption bands with a maximum wavelength of 401, 405, and 425 nm are assigned to the plasmon resonance of metallic AgNPs, indicating the existence of monodispersed spherical silver nanoparticles in the colloidal solution. Several research groups observed the surface plasmon resonance absorption band for AgNPs synthesized by similar methods with different plant extracts between 400–430 nm [50,51]. In contrast, the observed broad absorption band in the UV region at λ_max_ around 200 nm could be assigned to the presence of different organic compounds adsorbed on the surface of the AgNPs during the preparation process, such as flavonoids, phenolic acids, unsaturated groups, and heteroatoms such as S, N, O [52,53]. It is worth mentioning that the dispersion of the AgNPs is stable and that further aggregation after one month or eight months of preparation (Figure 1a) does not affect the stability of the nanoparticles, since the redshift of the absorption bands in the UV-Vis spectra were very small. These results revealed the formation of very small silver nanoparticles during the preparation process [54,55] and coincided with the observation of Shankar et al. [56] on the small size of formed AgNPs using *Azadirachta indica* leaf broth and their stability for one month.

It is believed that the phloroglucinols (hyperforin, Adhyperforin), the naphthodianthrone hypericin, the flavonoids (rutin, quercetin, quercitrin, quercitrin-hydrate, hyperoside), the bioflavonoid biapigenin, and the phenolic acid chlorogenic acid identified previously in the aqueous St John’s wort extract [46] played a role in the silver reduction and that the stabilization of the formed nanoelement might endow the AgNPs with advantageous bioactivities, such as antibacterial, antioxidant, and anti-cancer activity. Such an assumption is reinforced by the finding of Pradeep et al. [45], where the polar fraction containing phenolic acids and flavonoids was involved in the reduction of Ag+ ions, while the lower polar one containing biflavonoids, phloroglucinols, and naphthodianthrones acted as a capping agent. Moreover, in the same context, the presence of hypericin, pseudohypericin, hyperforin, and flavonoids in *H. perforatum* extracts and low polar compounds on the surface of NPs contributes to their antioxidant and antibacterial activities [39,40,41,57].

The dynamic light scattering technique was used to estimate the mean size, size distribution, and agglomeration potential of AgNPs [58]. Figure 1b shows the size of the resulting AgNPs depending on the relative frequency number weight (%). The hydrodynamic diameter distribution of green synthesized AgNPs in colloidal solution was found in the range of 15–30 nm with a polydispersity index (PDI) of 0.19. The zeta potential measurements showed that AgNPs carry a negative surface charge of −19 mv value in the colloidal solution (Figure 1c). The negatively charged surfaces of AgNPs might be attributed to their decoration with phenolic compounds, carboxylated polysaccharides, and the side chain of some amino acid residues [59,60], a charge that leads to less agglomeration and better physical stability. It is documented that the nanoparticles are well stabilized in colloidal solutions when the stabilizer on their surface is well adsorbed [61]. The dissociation of some H^+^ and total protonation of the hydroxyl groups of phytochemicals favor the repulsion between the nanoparticles, which discourages their aggregation.

The ATR-IR diagram shown in Figure 1d confirmed the existence of St. John’s wort extracted compounds on the surfaces of silver nanoparticles as a reducer and stabilizer. The presence of moieties from Naphthodianthrone, Phloroglucinol, flavonoids, and bioflavonoids derivatives, identified in our previous report on St. John’s wort extract [46], cause the appearance of well-known signals in the infrared region of the electromagnetic spectrum such as hydroxyl groups, alkyl moiety, and aromatic rings. Indeed, the positions of these bands in the ATR-FTIR spectra are considered a sensitive indicator of the conformational changes in the structure of the plant compounds due to their adsorption on the surfaces of silver nanoparticles [62]. The band appears in the region 675–900 cm^−1^ corresponding to C-H aromatic out of the plane [63]. The weak bands at 1230, 1242, and 1251 cm^−1^ correspond to C-O-C stretching in aromatic rings [64]. Both peaks at around 1385 and 1445 cm^−1^ are responsible for C-H stretching [65]. The peak at 1527 cm^−1^ is a response to stretching OH and C=C aromatic. The bands near 1617 cm^−1^ (sometimes 1640 cm^−1^) and near 1734 cm^−1^ refer to C=O, C-H, and C=O stretching in the aromatic ring. Bands in the range of 1923–2000 cm^−1^ prove the existence of an aromatic ring, while bands in the range of 2900–2990 cm^−1^ correspond to C-H stretching and the broad and intense band near 3400 cm^−1^ indicates the presence of O−H stretching in phenols [64,66,67]. The bands at 510 cm^−1^ and 590 cm^−1^ are attributed to the metal-ligand frequency stretching which formed due to the interaction between biomolecules and the AgNPs surfaces [68,69].

The XRD pattern shown in Figure 1e proves the formation of the nanocrystal structure of the green synthesized silver nanoparticle. The four special diffraction peaks at 2θ values 38.12°, 46.26°, 64.45°, and 77.38° which correspond to the planes (111), (200), (220), and (311), respectively, possess well-defined characteristic peaks corresponding to the face-centered cubic structure of pure metallic silver (JCPDS file No. 04-0783) [70,71]. The ratio between the intensities of the diffraction peaks of planes (200) and (111) is much lower than the typical value (0.52), revealing that the (111) plane is the predominant orientation [72], while the unassigned diffraction peaks can be related to the formation of the calcium nanocomposites [73]. In a similar study, the formation of calcium nanocomposite was observed during the biosynthesis of AgNPs by using *Carduus crispus* extract [74]. It is worth mentioning that the elemental analysis for *Hypericum perforatum* revealed that calcium exists in the highest concentration (5600 μg/g in leaves and 2500 μg/g in flowers) compared to the other elements in this plant [75], which indicates that the source of the calcium in our sample originated from the plant extract.

The broadening of the diffraction pattern peaks indicates the small average crystallite sizes of AgNPs, which can be measured using Debye-Scherrer’s equation [76]. The average size of crystalline silver nanoparticles was found to be around 12 nm.

The TGA analysis of phytochemical-coated AgNPs at 25 to 1000 °C with a heating rate of 1 °C min is presented in Figure 1f, and revealed three decomposition stages; accumulated water molecules absorbed on the surface of the nanoparticles are lost in the first stage after heating to 100 °C, the temperature was raised from 100 °C to 160 °C in the second stage, and heating was extended up to 600 °C in the third stage. The initial thermal deterioration of all the phytochemicals on the surface of these nanoparticles occurred and then the complete degradation continued. Finally, the ash and the mineral component remained due to the use of an inert atmosphere [77,78].

**Figure 2:**
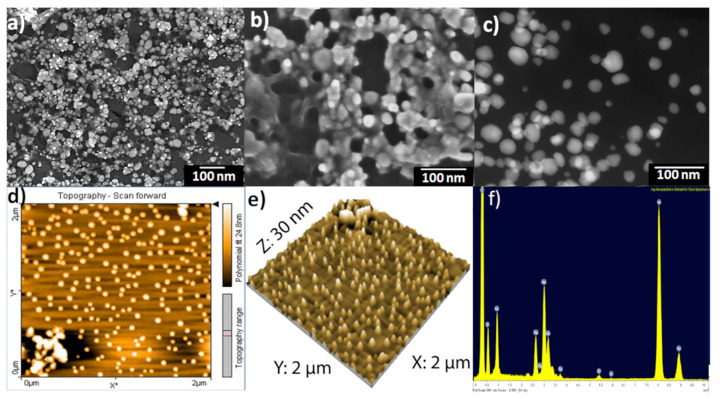
SEM images of *Hypericum perforatum* L. phytochemicals-capped AgNPs (**a**,**b**); scanning transmission electron microscopy dark-field (STEM-DF) image (**c**); AFM image of phytochemicals-capped AgNPs colloidal deposited as a film on glass slide by spin coating, scan scale: 2 µm × 2 µm, the color bar shows the scale in Z-direction (**d**); 2 µm × 2 µm AFM topography image of phytochemicals-capped AgNPs displayed as a three-dimensional projection (**e**) and quantitative results of energy dispersive X-ray (EDX) for phytochemicals-capped AgNPs (**f**).

The scanning electron microscopy (SEM) images of phytochemical coated AgNPs showed that most of the silver nanoparticles were spherical and monodisperse with sizes ranging from 20–40 nm, while other particles were aggregated with each other without a specific shape. This aggregation is a result of the network of organic compounds from St. John’s wort extract present as a capping protective layer on the surfaces of the silver nanoparticles (Figure 2a,b). Moreover, scanning transmission electron microscopy dark-field (STEM-DF) images (Figure 2c) of green synthesized silver nanoparticles confirmed that the particles’ sizes were about 20–40 nm and they were spherical and monodispersed.

The morphology and surface roughness of AgNPs was confirmed by atomic force microscopy (AFM). The magnification in particle size complexity is proportional to the size of the AFM tip and in sample preparation [79,80]. Figure 2d,e revealed spherical silver nanoparticles with grain diameters ranging from 20–40 nm, which agreed with those obtained from the DLS results. Larger scans screening a few micrometers resulted in a maximum peak-to-valley distance of 30 nm and a root mean square (RMS) roughness of 10 nm.

Figure 2f revealed the relative amounts of the elements in prepared AgNPs. EDX diagrams recorded specific elemental silver peaks at 2.8 keV, 3.5 keV, and 3.8 keV identical to the binding energies of Ag-L, Ag-M. This confirmed the existence of elemental silver in the resulting final colloidal solutions. The presence of the carbon and oxygen peaks is related to the organic layer from the St John’s wort plant, which is present on the surfaces of nanoparticles as a protective agent. As shown in Figure 2f, the relative amounts of carbon are much larger than others because of the carbon from the holder.

**Figure 3:**
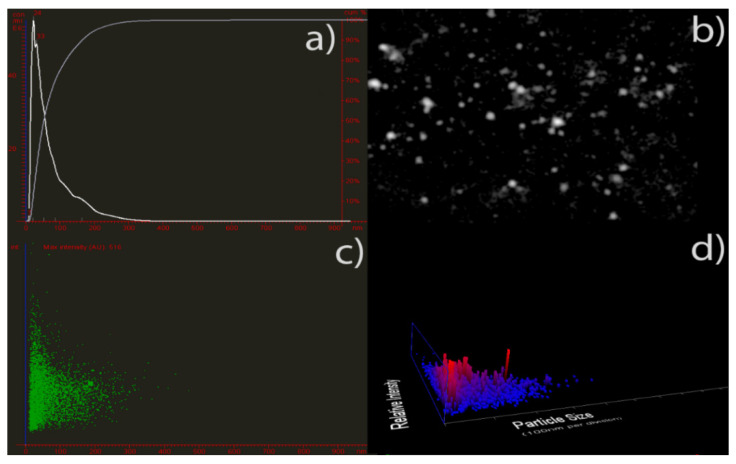
Nanoparticle tracking analysis of phytochemicals-capped AgNPs. Particle size vs. the number of particles (**a**); representative nanoparticle tracking analysis video frame (**b**), particle size vs. relative intensity (**c**), and the number of particles vs. particle size and light scattering intensity (3D plot) are shown in (**d**).

Moreover, the average diameter of AgNPs in the colloidal solution was determined by the nanoparticle tracking analysis (NTA), an analytical technique that is considered more precise than the dynamic light scattering method used to evaluate particle size distribution [81,82]. The particle size distribution calculated using this technique revealed monodisperse silver nanoparticles with an average diameter of 20–40 nm (Figure 3), which confirmed the DLS results.

### 3.2. Evaluation of Biological Activities of Hypericum perforatum Mediated AgNPs

**Figure 4:**
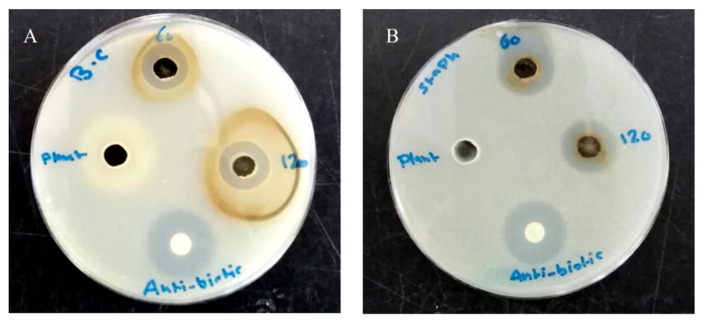
Photograph showing the antimicrobial activity of AgNPs against *B. cereus* (**A**) and *S. aureus* (**B**).

AgNPs revealed a broad-spectrum antibacterial activity against Gram-positive and negative tested bacterial strains, except for both *E. coli* strains that were resistant to the tested nanoparticles. The mean diameters of inhibition zones and the MIC values are presented in Table 1. According to the measured inhibition zones, Gram-positive bacteria were more susceptible to AgNPs than the Gram-negative ones in a concentration-dependent manner with less effectiveness against *B. subtilis* among other Gram-positive species; they caused the formation of 13.3–32 mm inhibition zones in Gram-positive bacteria and 13–19.7 mm in *P. aeruginosa* and *K. pneumonoiae* at the highest tested concentration (100 µg/well). The concentration required to cause the absence of growth in sensitive bacterial strains ranged from 6.25–12.5 µg/mL (MIC), with a bactericidal effect in most. Intriguingly, the plant extract did not cause inhibition in the growth of all tested bacterial strains. Figure 4 reveals the effect of AgNPs and plant extract on the growth of *S. aureus* and *B. cereus* in the agar diffusion test.

The ineffectiveness of *H. perforatum* aqueous extract to inhibit any of the tested bacterial strains concurred with previous reports on the inability of the plant leaf extract from *H. perforatum* [42] and different part extracts of *Carduus crispus* [74] to cause bacterial growth inhibition. Moreover, the antibacterial activity of AgNPs toward both Gram types of bacteria in the present study was in line with the previously reported broad-spectrum antibacterial activities of the nanoparticles in several studies, and was due to their ability to target and affect more than one cellular site and modulate the intracellular signaling and physiological pathways within the cell leading to their death [20,83]. In similar studies, AgNPs biosynthesized using extracts of *H. perforatum* [41,42], *Hypericum hookerianum* [84], *Hypericum scabrum* [85], *Origanum vulgare* [86], and *Carduus crispus* [74] exhibited activity against both Gram-negative and Gram-positive bacteria.

Also in line with our results, Gram-positive bacteria were documented to be slightly more sensitive than the negative ones to the tested nanoparticles [87,88,89,90]. Controversial results were obtained by other studies that reveal more sensitivity of Gram-negative bacteria than Gram-positive ones toward tested AgNPs [86,91,92]. Interestingly, it was postulated that Ag^+^ ions from the AgNPs have different targets between Gram-negative and positive bacteria [93], though the exhibited activity of AgNPs synthesized by *Carduus crispus* on both Gram-types of bacteria was not affected by the difference in the bacterial wall [92].

**Figure 5:**
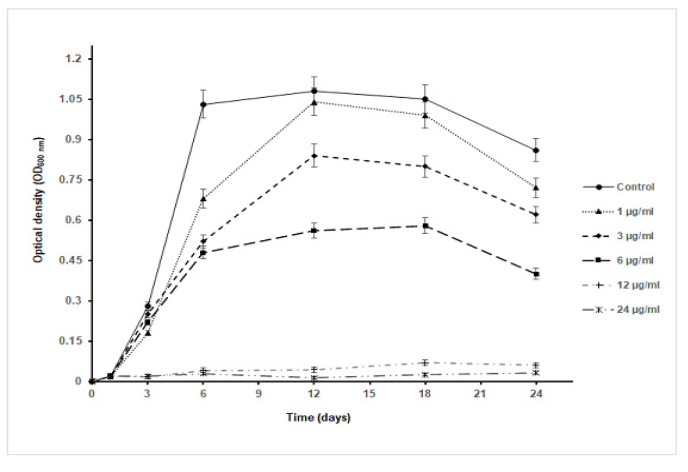
Growth curve of *S. aureus* in Muller Hinton broth containing different concentrations of AgNPs.

**Figure 6:**
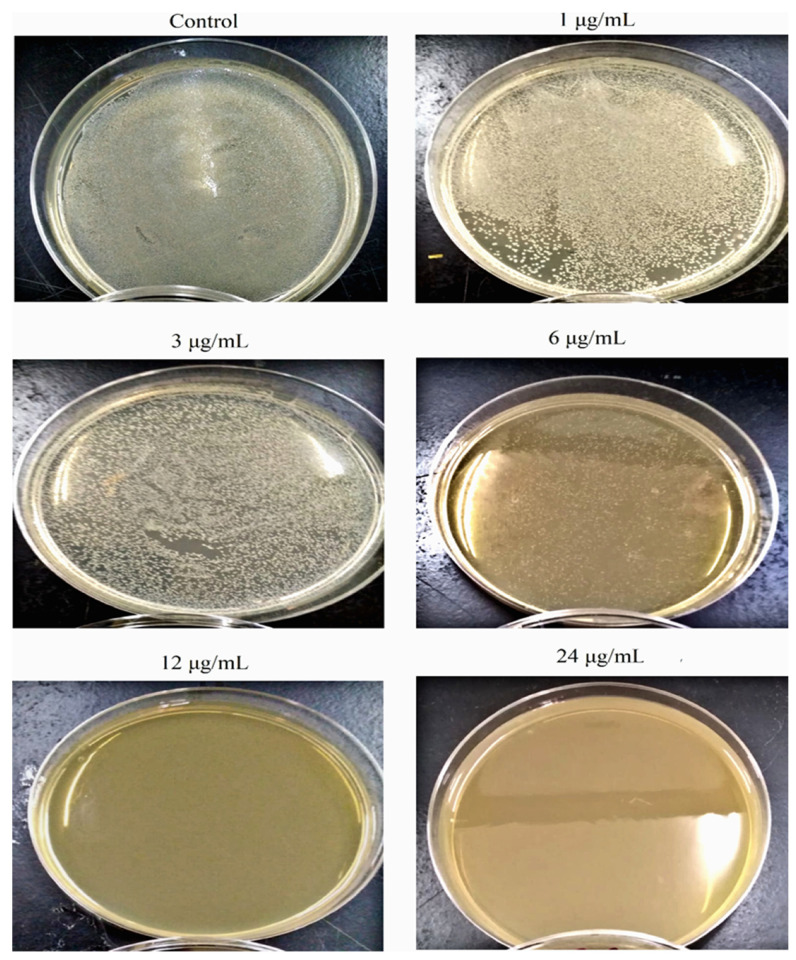
Photograph of *S. aureus* grown on Muller Hinton agar as a function of AgNPs concentrations.

As *S. aureus* was the most susceptible bacterial test strain to AgNPs, it was selected to estimate the effect of nanoparticles on the growth kinetics of the bacterium (Figure 5). As the concentration of the nanoparticle increases, a decrease in the OD_600nm_ value was noticed in comparison to the control culture and at different time intervals of sampling. Remarkably, the maximum cell density attained was about 50% of that reached by the control culture as the bacterium was exposed to almost ½ MIC concentration (6 µg/mL). Furthermore, at 12 µg/mL and 24 µg/mL, the growth of *S. aureus* was inhibited with an undetectable increase in the OD_600nm_ value throughout the whole period of cultivation (24 h). Such a finding indicated that the AgNPs have a toxic effect on the tested bacterium and their toxicity increase at higher concentrations of the nanoparticles. Intriguingly, the specific growth rate of *S. aureus* in the presence of AgNPs declined with an increase in the tested nanoparticle concentrations to 6 µg/mL; at concentrations over 6 µg/mL, the specific growth rate was almost zero, highlighting the higher toxicity of the nanoparticles on the bacterium (Table 2). The specific growth rate was decreased from 0.43 h^−1^ in the control culture to 0.10 h^−1^ in the culture exposed to 6 µg/mL AgNPs. In terms of CFU, as the concentration of nanoparticles increased in Muller Hinton agar plates, there was a decrease in the number of growing colonies that were absent starting from concentrations ≥12 µg/mL, indicating a concentration-dependent antibacterial activity of AgNPs (Figure 6).

**Figure 7:**
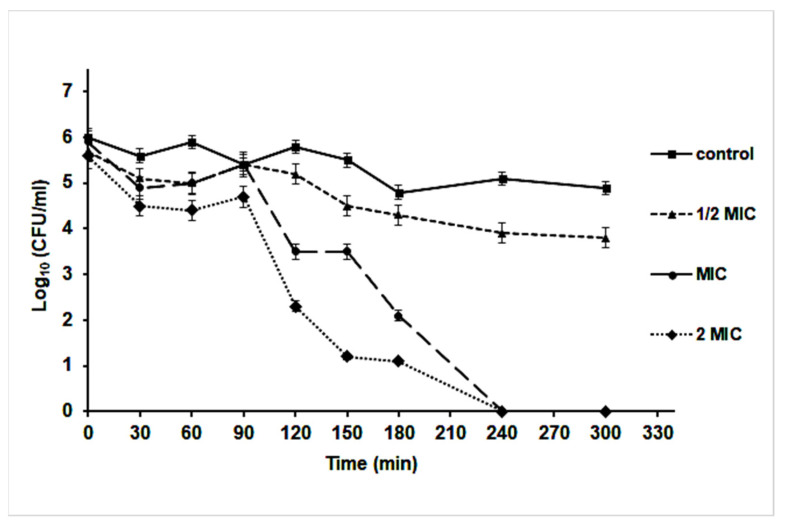
Time-kill curve of *S. aureus* in Muller Hinton broth medium supplemented with different concentrations of AgNPs.

A time-kill assay revealed that the effect of nanoparticles started after 90 min of incubation at different MIC concentrations. Interestingly, at ½ MIC concentration, the Log_10_ (CFU/mL) was decreased by 10% compared to the control culture (without AgNPs). However, an almost 10^3^-fold reduction in CFU/mL of *S. aureus* was noticed after 120 min of cultivation at MIC and 2 MIC concentrations of AgNPs, which indicated a bactericidal effect of the nanoparticles; it caused the killing of the bacterium after 240 min of cultivation (Figure 7).

It is worth noting that the promising antibacterial activity of AgNPs was attributed to their ability to destroy cell membranes, cause intracellular damage, alter genetic material, and cause oxidative stress in the bacterial cell [94]. Due to their size, nanoparticles have a large surface area that enables them to attach to the cell wall, penetrate the cell, and cause a disturbance in the membrane permeability, leading to leakage of the cell content [95,96]. Interestingly, the capping of the nanoparticles with moieties of the lipophilic hypericin, hyperforin, and adhyperforin might facilitate their diffusion across the hydrophobic cell membrane of the bacterial cells; meanwhile, the presence of moieties from the flavonoid and phenols such as rutin and quercitrin surrounding their surfaces could increase their capacity to form complexes with extracellular and soluble proteins and with the cell wall [97]. These chemical constituents were detected among others in the extract of the St. John’s wort in a previous report [46]. Moreover, AgNPs can bind to sulfur and phosphorus-containing proteins leading to protein and nucleic acids inactivation [98]. Furthermore, Ag+ ions, released through the oxidation dissolution process, alter the respiratory chain and facilitate the generation of reactive oxygen species (ROS) via their interaction with thiol groups of various enzymes and proteins, thus causing cell death through the inactivation of DNA replication, ATP production, and the activation of the apoptosis pathway [99,100,101].

**Figure 8:**
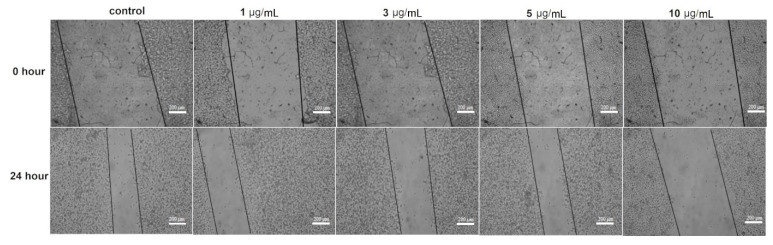
Effect of different AgNPs concentrations on the wound contraction in the fibroblast cell line.

The effect of the AgNPs on the fibroblast cell migration was examined by the wound migration (contraction) method. The nanoparticles at concentrations of 1–5 µg/mL caused a 52.2–55.5% contraction in the wound distance compared to 67.5% wound closure in untreated fibroblast cells (control) after 24 h of treatment (Figure 8). However, 31.6% of the wound distance was contracted at 10 µg/mL of AgNPs, a concentration around the IC_50_ of the nanoparticle to the fibroblast cell line (data not shown); the decrease in the wound closure at this concentration compared to other tested doses was due to the cytotoxic effect of the nanoparticles on the tested fibroblast cells.

**Figure 9:**
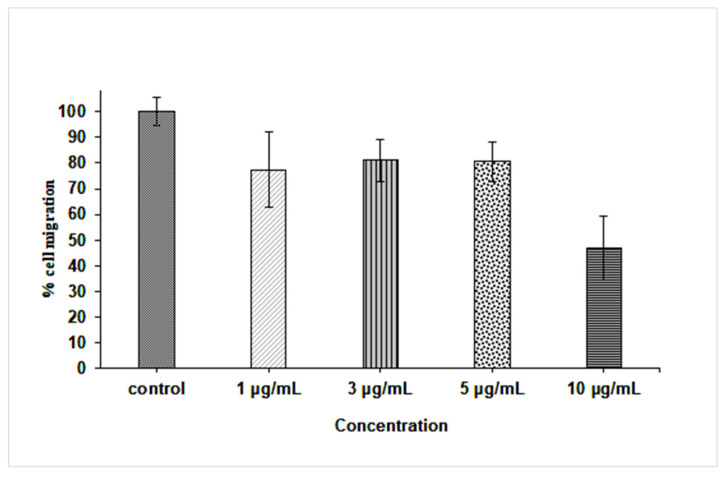
Percentage of fibroblast cell migration after 24 h treatment with 0, 1, 3, 5, and 10 µg/mL AgNPs.

In fact, wound migration was non-significantly inhibited when fibroblast cells were treated with 1–5 µg/mL AgNPs (inhibition 19–23% compared to control), but it was significantly inhibited at 10 µg/mL (inhibition 46.8%, *p* < 0.5) (Figure 9). Fibroblast cells play a fundamental role in body homeostasis through the secretion of growth factors, the release of matrix metalloproteinases, collagen expression, and facilitating contraction of healing wounds [102]. Some studies had reported that ionization of AgNPs by reacting with free oxygen produces a reactive Ag which might regulate the transcriptional factor FOXO1 that stimulates the wound healing molecule, TGF-β1 [89]. Moreover, AgNPs were able to promote fibroblast differentiation and wound contraction in ex vivo systems using rodent models; the ability of these nanoparticles to exert their wound healing effect is through the modulation of inflammatory cytokines [103,104,105].

On the contrary, in the current study, it was noticed that the nanoparticles at low concentrations (<IC50) caused an inhibition in fibroblast cell migration and wound contraction; a finding that coincided with the reported effect of the AgNPs in the negative modulation of extracellular matrix molecules (ECM) and laminin deposition, the imbalanced expression of integrins, the impaired reorganization or F-actin cytoskeleton, and decreased human fibroblast migration [102]. Similarly, it was reported that AgNPs-based dressings were unable to reestablish normal skin formation, as they did not cause fibroblast and keratinocyte proliferation [106]. However, *Curcuma longa* capped AgNPs coating cotton fabrics were effective in wound healing in the fibroblast (L929) cell lines in vitro [13], though such activity was lower than that of untreated control cells. These results revealed that the ability of AgNPs in promoting wound healing depends on the test system employed, in vivo or in vitro, as well as the presence of bio-factors such as growth factors and cytokines.

Altogether, smaller AgNPs than previously prepared [46] were synthesized to get a large surface-to-volume ratio. They were functionalized with moieties from *H. perforatum* aqueous extract that influence NPs’ surface charge, hydrophobicity, and/or solubility, thus influencing the particles’ structure, size, shape, morphology, degree of aggregation, particle dissolution, ion release, and finally the potential bioactivity. More studies are required in order to confirm the potential role of AgNPs in the wound healing process, and their health impact should be critically evaluated.

## 4. Conclusions

During the current study, the aqueous extract of the St. John’s wort plant was used to synthesize silver nanoparticles as an environmentally friendly, fast, and clean method. The synthesized AgNPs were characterized by UV-VIS, DLS, zeta potential, FTIR, XRD, SEM, TEM, TGA, and NTA. The resulting AgNPs were spherical and monodisperse, with face-centered cubic crystal structures, and 20–40 nm in size. FTIR revealed the capping of AgNPs with functional groups of St. John’s wort extract. The green synthesized nanoparticles exhibited a broad spectral antibacterial activity with Gram-positive test strains as the most susceptible group. The presence of the plant functional group decorating the nanoparticles’ surfaces might facilitate their binding to cellular targets and exert their activities. Their bactericidal effect on *S. aureus* was noticed after 120 min of cultivation at MIC and 2 MIC concentrations; the bacterial specific growth rate significantly declined as the concentration of the tested nanoparticles increased up to ½ MIC, and it was zero at MIC and 2 MIC. Furthermore, the wound migration (closure) did not affect AgNPs treatments up to 10 µg/mL.

## Figures and Tables

**Table 1:** Antibacterial activity of AgNPs and positive control against tested bacterial strains.

Bacterial Strain	Inhibition Zone (mm ± SD) (µg/Well)		S (10 µg/Disc)	MIC (µg/mL)	
	50	100		AgNPs	S
**Gram-positive**					
*B. subtilis*	11.7 ± 0.6	13.3 ± 0.6	23.3 ± 1.6	12.5 c	0.63 s
*B. cereus*	19.0 ± 1	24.3 ± 2.1	29.7 ± 1.3	6.25 s	0.31 s
*S. aureus*	27.7 ± 1.5	32.0 ± 1.3	31.3 ± 0.7	12.5 c	0.63 c
**Gram-negative**					
*E. coli*	NA	NA	18.0 ± 1.3	>100	5 c
*E. coli* (clinical)	NA	NA	12.3 ± 0.7	>100	>10
*K. pneumonia* (clinical)	10.3 ± 0.6	13 ± 1	15.7 ± 0.7	12.5 c	10 c
*P. aeruginosa*	16.7 ± 1.5	19.7 ± 1.1	17.1 ± 0.3	6.25 s	2.5 s

S: streptomycin; s: biostatic; c: biocidal; NA: not active.

**Table 2:** The effect of AgNPs on the growth kinetics of *S. aureus* tested bacterial strains.

Concentration (µg/mL)	Specific Growth Rate (h^−1^)	Maximum Growth Intensity (OD_600nm_)
Control	0.43 ± 0.027	1.08 ± 0.05
1	0.20 ± 0.015	1.04 ± 0.07
3	0.13 ± 0.008	0.84 ± 0.04
6	0.10 ± 0.011	0.58 ± 0.03
12	-	0.07 ± 0.01
24	-	0.032 ± 0.007

## Data Availability

The data presented in this study are available on request from the corresponding author.

## References

[1] Khurana A., Tekula S., Saifi M.A., Venkatesh P., Godugu C. (2019). Therapeutic applications of selenium nanoparticles. Biomed. Pharmacother..

[2] Ikram M., Javed B., Raja N.I., Mashwani Z.U.R. (2021). Biomedical potential of plant-based selenium nanoparticles: A comprehensive review on therapeutic and mechanistic aspects. Int. J. Nanomed..

[3] Chinnasamy G., Chandrasekharan S., Koh T.W., Bhatnagar S. (2021). Synthesis, characterization, antibacterial and wound healing efficacy of silver nanoparticles from *Azadirachta indica*. Front. Microbiol..

[4] Alahmad A., Feldhoff A., Bigall N.C., Rusch P., Scheper T., Walter J.G. (2021). *Hypericum perforatum* L.-mediated green synthesis of silver nanoparticles exhibiting antioxidant and anticancer activities. Nanomaterials.

[5] Akintelu S.A., Bo Y., Folorunso A.S. (2020). A review on synthesis, optimization, mechanism, characterization, and antibacterial application of silver nanoparticles synthesized from plants. J. Chem..

[6] El-Seedi H.R., El-Shabasy R.M., Khalifa S.A.M., Saeed A., Shah A., Shah R., Iftikhar F.J., Abdel-Daim M.M., Omri A., Hajrahand N.H. (2019). Metal nanoparticles fabricated by green chemistry using natural extracts: Biosynthesis, mechanisms, and applications. RSC Adv..

[7] Chandran S.P., Chaudhary M., Pasricha R., Ahmad A., Sastry M. (2006). Synthesis of gold nanotriangles and silver nanoparticles using *Aloe vera* plant extract. Biotechnol. Prog..

[8] Li S., Shen Y., Xie A., Yu X., Qiu L., Zhang L., Zhang Q. (2007). Green synthesis of silver nanoparticles using *Capsicum annuum* L. extract. Green Chem..

[9] Elumalai E., Prasad T., Hemachandran J., Therasa S.V., Thirumalai T., David E. (2010). Extracellular synthesis of silver nanoparticles using leaves of *Euphorbia hirta* and their antibacterial activities. J. Pharm. Sci. Res..

[10] Krishnaraj C., Jagan E., Rajasekar S., Selvakumar P., Kalaichelvan P., Mohan N. (2010). Synthesis of silver nanoparticles using *Acalypha indica* leaf extracts and its antibacterial activity against water borne pathogens. Colloids Surf. B Biointerfaces.

[11] Veerasamy R., Xin T.Z., Gunasagaran S., Xiang T.F.W., Yang E.F.C., Jeyakumar N., Dhanaraj S.A. (2011). Biosynthesis of silver nanoparticles using mangosteen leaf extract and evaluation of their antimicrobial activities. J. Saudi Chem. Soc..

[12] Khan S.A., Shahid S., Lee C.-S. (2020). Green synthesis of gold and silver nanoparticles using leaf extract of *Clerodendrum inerme*; characterization, antimicrobial, and antioxidant activities. Biomolecules.

[13] Maghimaa M., Alharbi S.A. (2020). Green synthesis of silver nanoparticles from *Curcuma longa* L. and coating on the cotton fabrics for antimicrobial applications and wound healing activity. J. Photochem. Photobiol. B Biol..

[14] Nilavukkarasi M., Vijayakumar S., Prathip Kumar S. (2020). Biological synthesis and characterization of silver nanoparticles with *Capparis zeylanica* L. leaf extract for potent antimicrobial and anti proliferation efficiency. Mater. Sci. Energy Technol..

[15] Olfati A., Kahrizi D., Balaky S.T.J., Sharifi R., Tahir M.B., Darvishi E. (2021). Green synthesis of nanoparticles using *Calendula officinalis* extract from silver sulfate and their antibacterial effects on *Pectobacterium caratovorum*. Inorg. Chem. Commun..

[16] Jain D., Daima H.K., Kachhwaha S., Kothari S. (2009). Synthesis of plant-mediated silver nanoparticles using papaya fruit extract and evaluation of their antimicrobial activities. Dig. J. Nanomater. Biostruct..

[17] Bar H., Bhui D.K., Sahoo G.P., Sarkar P., De S.P., Misra A. (2009). Green synthesis of silver nanoparticles using latex of *Jatropha curcas*. Colloids Surf. A Physicochem. Eng. Asp..

[18] Sathishkumar M., Sneha K., Won S.W., Cho C.W., Kim S., Yun Y.S. (2009). *Cinnamon zeylanicum* bark extract and powder mediated green synthesis of nano-crystalline silver particles and its bactericidal activity. Colloids Surf. B Biointerfaces.

[19] Loo Y.Y., Rukayadi Y., Nor-Khaizura M.-A.-R., Kuan C.H., Chieng B.W., Nishibuchi M., Radu S. (2018). In vitro antimicrobial activity of green synthesized silver nanoparticles against selected Gram-negative foodborne pathogens. Front. Microbiol..

[20] Paladini F., Pollini M. (2019). Antimicrobial silver nanoparticles for wound healing application: Progress and future trends. Materials.

[21] Yuwen L., Sun Y., Tan G., Xiu W., Zhang Y., Weng L., Teng Z., Wang L. (2018). MoS2@polydopamine-Ag nanosheets with enhanced antibacterial activity for effective treatment of *Staphylococcus aureus* biofilms and wound infection. Nanoscale.

[22] Silvero C.M.J., Rocca D.M., de la Villarmois E.A., Fournier K., Lanterna A.E., Perez M.F., Becerra M.C., Scaiano J.C. (2018). Selective photoinduced antibacterial activity of amoxicillin-coated gold nanoparticles: From one-step synthesis to in vivo cytocompatibility. ACS Omega.

[23] Dhas T.S., Kumar V.G., Karthick V.A., Angel K.J., Govindaraju K. (2014). Facile synthesis of silver chloride nanoparticles using marine alga and its antibacterial efficacy. Spectrochim. Acta Part A Mol. Biomol. Spectrosc..

[24] Das C.A., Kumar V.G., Dhas T.S., Karthick V., Govindaraju K., Joselin J.M., Baalamurugan J. (2020). Antibacterial activity of silver nanoparticles (biosynthesis): A short review on recent advances. Biocatal. Agric. Biotechnol..

[25] Shanmuganathan R., Karuppusamy I., Saravanan M., Muthukumar H., Ponnuchamy K., Ramkumar V.S., Pugazhendhi A. (2019). Synthesis of silver nanoparticles and their biomedical applications—A comprehensive review. Curr. Pharm. Des..

[26] Kailasa S.K., Park T.J., Rohit J.V., Koduru J.R., Grumezescu A.M. (2019). Antimicrobial activity of silver nanoparticles. Nanoparticles in Pharmacotherapy.

[27] Wei L., Lu J., Xu H., Patel A., Chen Z.S., Chen G. (2015). Silver nanoparticles: Synthesis, properties, and therapeutic applications. Drug Discov. Today.

[28] Burdușel A.C., Gherasim O., Grumezescu A.M., Mogoantă L., Ficai A., Andronescu E. (2018). Biomedical applications of silver nanoparticles: An up-to-date overview. Nanomaterials.

[29] Almatroudi A. (2020). Silver nanoparticles: Synthesis, characterisation and biomedical applications. Open Life Sci..

[30] Lenard J., Rabson A., Vanderoef R. (1993). Photodynamic inactivation of infectivity of human immunodeficiency virus and other enveloped viruses using hypericin and rose bengal: Inhibition of fusion and syncytia formation. Proc. Natl. Acad. Sci. USA.

[31] Davidson J.R.T., Connor K.M. (2001). John’s wort in generalized anxiety disorder: Three case reports. J. Clin. Psychopharmacol..

[32] Bukhari I.A., Dar A., Khan R.A. (2004). Antinociceptive activity of methanolic extracts of St. John’s Wort (*Hypericum perforatum*) preparation. Pak. J. Pharm. Sci..

[33] Schepetkin I.A., Özek G., Özek T., Kirpotina L.N., Khlebnikov A.I., Quinn M.T. (2020). Chemical composition and immunomodulatory activity of *Hypericum perforatum* essential oils. Biomolecules.

[34] Shafaghat A. (2011). Antioxidant, antimicrobial activities and fatty acid components of flower, leaf, stem and seed of *Hypericum scabrum*. Nat. Prod. Commun..

[35] Derun E.M., Eslek Z., Piskin S. (2013). Evaluation of analysis on the extracts from *Hypericum perforatum* L. grown in Turkey. Int. J. Chem. Nucl. Metall. Mater. Eng..

[36] Upton R., Upton R., Pharmacopoeia A.H., Cott J., Williamson E., St. Graff A. (1997). John’s Wort: Hypericum Perforatum: Quality Control, Analytical and Therapeutic Monograph.

[37] Lavie G., Mazur Y., Lavie D., Prince A., Pascual D., Liebes L., Levin B., Meruelo D. (1995). Hypericin as an inactivator of infectious viruses in blood components. Transfusion.

[38] Lakmann G., Schule C., Baghai T., Kieser M. (1998). St. John’s Wort in mild to moderate depression: The relevance of hyperforin for the clinical efficacy. Pharmacopsychiatry.

[39] Zou Y., Lu Y., Wei D. (2004). Antioxidant activity of a flavonoid-rich extract of *Hypericum perforatum* L. in vitro. J. Agric. Food Chem..

[40] Saddiqe Z., Naeem I., Maimoona A. (2010). A review of the antibacterial activity of *Hypericum perforatum* L.. J. Ethnopharmacol..

[41] Gitea D., Teodorescu A., Pantis C., Tit D.M., Bungau A.F., Bogdan M., Fodor I.K., Bustea C. (2020). Green synthesis of silver nanoparticles using *Hypericum perforatum* L. extract and evaluation of their antibacterial activity. Rev. Chim..

[42] Ozgen A., Bilgic E., Aydin S.G., Nizamlioglu M. (2019). Characterization of biosynthesized silver nanoparticles using *Hypericum perforatum* leaf and determination of their antibacterial activity. Med. Sci..

[43] Prakash D.J., Arulkumar S., Sabesan M. (2010). Effect of nanohypericum (*Hypericum perforatum* gold nanoparticles) treatment on restraint stress-induced behavioral and biochemical alteration in male albino mice. Pharmacogn. Res..

[44] Jafarizad A., Safaee K., Vahid B., Khataee A., Ekinci D. (2019). Synthesis and characterization of gold nanoparticles using *Hypericum perforatum* and *Nettle* aqueous extracts: A comparison with turkevich method. Environ. Prog. Sustain. Energy.

[45] Pradeep M., Kruszka D., Kachlicki P., Mondal D., Franklin G. (2022). Uncovering the phytochemical basis and the mechanism of plant extract-mediated eco-friendly synthesis of silver nanoparticles using ultra-performance liquid chromatography coupled with a photodiode array and high-resolution mass spectrometry. ACS Sustain. Chem. Eng..

[46] Alahmad A., Alghoraibi I., Zein R., Kraft S., Dräger G., Walter J., Scheper T. (2022). Identification of major constituents of *Hypericum perforatum* L. extracts in Syria by development of a rapid, simple and reproducible HPLC-ESI-Q-TOF MS analysis and their antioxidant activities. ACS Omega.

[47] Al-Zereini W.A. (2014). Bioactive crude extracts from four bacterial isolates of marine sediments from Red Sea, Gulf of Aqaba, Jordan. Jord. J. Biol. Sci..

[48] CLSI (2012). Performance Standards for Antimicrobial Susceptibility Testing.

[49] Wadhwani S., Gorain M., Banerjee P., Shedbalkar U., Singh R., Kundu G., Chopade B. (2017). Green synthesis of selenium nanoparticles using *Acinetobacter* sp. SW30: Optimization, characterization and its anticancer activity in breast cancer cells. Int. J. Nanomed..

[50] Ma L., Su W., Liu J.X., Zeng X.X., Huang Z., Li W., Liu Z.C., Tang J.X. (2017). Optimization for extracellular biosynthesis of silver nanoparticles by *Penicillium aculeatum* Su1 and their antimicrobial activity and cytotoxic effect compared with silver ions. Mater. Sci. Eng. C Mater. Biol. Appl..

[51] Rolim W.R., Pelegrino M.T., de Araújo Lima B., Ferraz L.S., Costa F.N., Bernardes J.S., Rodigues T., Brocchi M., Seabra A.B. (2019). Green tea extract mediated biogenic synthesis of silver nanoparticles: Characterization, cytotoxicity evaluation and antibacterial activity. Appl. Surf. Sci..

[52] Ernawati, Suprayitno E., Hardoko, Yanuhar U. (2019). Extraction of bioactive compounds fruit from *Rhizophora mucronata* using sonication method. IOP Conf. Ser. Earth Environ. Sci..

[53] Jarzębski M., Smułek W., Baranowska H.M., Masewicz Ł., Kobus-Cisowska J., Ligaj M., Kaczorek E. (2020). Characterization of St. John’s wort (*Hypericum perforatum* L.) and the impact of filtration process on bioactive extracts incorporated into carbohydrate-based hydrogels. Food Hydrocoll..

[54] Alim-Al-Razy M., Asik Bayazid G.M., Rahman R.U., Bosu R., Shamma S.S. (2020). Silver nanoparticle synthesis, UV-Vis spectroscopy to find particle size and measure resistance of colloidal solution. J. Phys. Conf. Ser..

[55] Aziz S.B., Abdullah O.G., Saber D.R., Rasheed M.A., Ahmed H.M. (2017). Investigation of metallic silver nanoparticles through UV-Vis and optical micrograph techniques. Int. J. Electrochem. Sci..

[56] Shankar S.S., Rai A., Ahmad A., Sastry M. (2004). Rapid synthesis of Au, Ag, and bimetallic Au core–Ag shell nanoparticles using Neem (*Azadirachta indica*) leaf broth. J. Colloid Interface Sci..

[57] Marslin G., Selvakesavan R.K., Franklin G., Sarmento B., Dias A.C. (2015). Antimicrobial activity of cream incorporated with silver nanoparticles biosynthesized from *Withania somnifera*. Int. J. Nanomed..

[58] Gontijo L.A., Raphael E., Ferrari D.P., Ferrari J.L., Lyon J.P., Schiavon M.A. (2020). pH effect on the synthesis of different size silver nanoparticles evaluated by DLS and their size-dependent antimicrobial activity. Matéria (Rio Jan.).

[59] Gunti L., Dass R.S., Kalagatur N.K. (2019). Phytofabrication of selenium nanoparticles from *Emblica officinalis* fruit extract and exploring its biopotential applications: Antioxidant, antimicrobial, and biocompatibility. Front. Microbiol..

[60] Pyrzynska K., Sentkowska A. (2021). Biosynthesis of selenium nanoparticles using plant extracts. J. Nanostructure Chem..

[61] Chandran P.R., Naseer M., Udupa N., Sandhyarani N. (2012). Size controlled synthesis of biocompatible gold nanoparticles and their activity in the oxidation of NADH. Nanotechnology.

[62] Haroon H., Kulandhaivel M., Anbalagan S., Sankareswaran M., Abirami K., Prabhavathi P., Manikandan A. (2017). Green synthesis of silver nanoparticles using *Hybanthus enneaspermus* plant extract against nosocomial pathogens with nanofinished antimicrobial cotton fabric. Glob. J. Nanomed..

[63] Margoshes M., Fassel V.A. (1955). The infrared spectra of aromatic compounds: I. The out-of-plane C-H bending vibrations in the region 625–900 cm^−1^. Spectrochim. Acta.

[64] Lehto J., Louhelainen J., Kłosińska T., Drożdżek M., Alén R. (2018). Characterization of alkali-extracted wood by FTIR-ATR spectroscopy. Biomass Convers. Biorefin..

[65] Ashokkumar R., Ramaswamy M. (2014). Phytochemical screening by FTIR spectroscopic analysis of leaf extracts of selected Indian medicinal plants. Int. J. Curr. Microbiol. Appl. Sci..

[66] Petković M. (2012). O–H stretch in phenol and its hydrogen-bonded complexes: Band position and relaxation pathways. J. Phys. Chem. A.

[67] Brangule A., Šukele R., Bandere D. (2020). Herbal medicine characterization perspectives using advanced FTIR sample techniques—diffuse reflectance (DRIFT) and photoacoustic spectroscopy (PAS). Front. Plant Sci..

[68] Ahmad T., Wani I.A., Manzoor N., Ahmed J., Asiri A.M. (2013). Biosynthesis, structural characterization and antimicrobial activity of gold and silver nanoparticles. Colloids Surf. B Biointerfaces..

[69] Begam J.N. (2016). Biosynthesis and characterization of silver nanoparticles (AgNPs) using marine bacteria against certain human pathogens. Int. J. Adv. Sci. Res..

[70] Anigol L.B., Charantimath J.S., Gurubasavaraj P.M. (2017). Effect of concentration and pH on the size of silver nanoparticles synthesized by green chemistry. Org. Med. Chem. Int. J..

[71] Mondal A., Hajra A., Shaikh W.A., Chakraborty S., Mondal N.K. (2019). Synthesis of silver nanoparticle with *Colocasia esculenta* (L.) stem and its larvicidal activity against *Culex quinquefasciatus* and *Chironomus* sp.. Asian Pac. J. Trop. Biomed..

[72] Philip D. (2009). Biosynthesis of Au, Ag and Au–Ag nanoparticles using edible mushroom extract. Spectrochim. Acta Part A Mol. Biomol. Spectrosc..

[73] Vijai Anand K., Reshma M., Kannan M., Muthamil Selvan S., Chaturvedi S., Shalan A.E., Govindaraju K. (2021). Preparation and characterization of calcium oxide nanoparticles from marine molluscan shell waste as nutrient source for plant growth. J. Nanostructure Chem..

[74] Urnukhsaikhan E., Bold B.E., Gunbileg A., Sukhbaatar N., Mishig-Ochir T. (2021). Antibacterial activity and characteristics of silver nanoparticles biosynthesized from *Carduus crispus*. Sci. Rep..

[75] Dastagir G., Ahmed R., Shereen S. (2016). Elemental, nutritional, phytochemical and biological evaluation of Hypericum perforatum Linn. Pak. J. Pharm. Sci..

[76] Sagadevan S., Vennila S., Singh P., Lett J.A., Johan M.R., Muthiah B., Lakshmipathy M. (2019). Facile synthesis of silver nanoparticles using *Averrhoa bilimbi* L and Plum extracts and investigation on the synergistic bioactivity using in vitro models. Green Process. Synth..

[77] Ivashchenko O., Przysiecka Ł., Peplinska B., Flak D., Coy E., Jarek M., Zalewski T., Musiał A., Jurga S. (2021). Organic–inorganic hybrid nanoparticles synthesized with *Hypericum perforatum* extract: Potential agents for photodynamic therapy at ultra-low power light. ACS Sustain. Chem. Eng..

[78] Fernandes F.H., Santana C.P., Santos R.L., Correia L.P., Conceição M.M., Macêdo R.O., Medeiros A.C. (2013). Thermal characterization of dried extract of medicinal plant by DSC and analytical techniques. J. Therm. Anal. Calorim..

[79] Saware K., Sawle B., Salimath B., Jayanthi K., Abbaraju V. (2014). Biosynthesis and characterization of silver nanoparticles using *Ficus benghalensis* leaf extract. Int. J. Res. Eng. Technol..

[80] Menon S., Agarwal H., Kumar S.R., Kumar S.V. (2017). Green synthesis of silver nanoparticles using medicinal plant *Acalypha indica* leaf extracts and its application as an antioxidant and antimicrobial agent against foodborne pathogens. Int. J. Appl. Pharm..

[81] Filipe V., Hawe A., Jiskoot W. (2010). Critical evaluation of nanoparticle tracking analysis (NTA) by nanosight for the measurement of nanoparticles and protein aggregates. Pharm. Res..

[82] Fan Y., Sahdev P., Ochyl L.J., Akerberg J.J., Moon J.J. (2015). Cationic liposome-hyaluronic acid hybrid nanoparticles for intranasal vaccination with subunit antigens. J. Control. Release.

[83] Lee S.H., Jun B.-H. (2019). Silver nanoparticles: Synthesis and application for nanomedicine. Int. J. Mol. Sci..

[84] Manoj L., Vishwakarma V., Samal S.S., Seeni S. (2015). Green synthesis of silver nanoparticles using hypericin-rich shoot cultures of *Hypericum hookerianum* and evaluation of anti-bacterial activities. J. Exp. Nanosci..

[85] Nazari Z., Shafaghat A. (2017). Biological synthesis and antimicrobial activity of nano-silver using *Hypericum scabrum* seed extract. Inorg. Nano-Met. Chem..

[86] Shaik M.R., Khan M., Kuniyil M., Al-Warthan A., Alkhathlan H.Z., Siddiqui M.R.H., Shaik J.P., Ahamed A., Mahmood A., Khan M. (2018). Plant-extract-assisted green synthesis of silver nanoparticles using *Origanum vulgare* L. extract and their microbicidal activities. Sustainability.

[87] Rathnayake W.G.I.U., Ismail H., Baharin A., Darsanasiri A.G.N.D., Rajapakse S. (2012). Synthesis and characterization of nano-silver based natural rubber latex foam for imparting antibacterial and anti-fungal properties. Polym. Test..

[88] Padalia H., Moteriya P., Chanda S. (2015). Green synthesis of silver nanoparticles from marigold flower and its synergistic antimicrobial potential. Arab. J. Chem..

[89] Gomathi M., Rajkumar P.V., Prakasam A., Ravichandran K. (2017). Green synthesis of silver nanoparticles using *Datura stramonium* leaf extract and assessment of their antibacterial activity. Resour.-Effic. Technol..

[90] Khandel P., Shahi S.K., Soni D.K., Yadaw R.K., Kanwar L. (2018). *Alpinia calcarata*: Potential source for the fabrication of bioactive silver nanoparticles. Nano Converg..

[91] Kim J.S., Kuk E., Yu K.N., Kim J.H., Park S.J., Lee H.J., Kim S.H., Park Y.K., Park Y.H., Hwang C.Y. (2007). Antimicrobial effects of silver nanoparticles. Nanomed. Nanotechnol. Biol. Med..

[92] Ghetas H.A., Abdel-Razek N., Shakweer M.S., Abotaleb M.M., Paray B.A., Ali S., Eldessouki E.A., Dawood M.A., Khalil R.H. (2022). Antimicrobial activity of chemically and biologically synthesized silver nanoparticles against some fish pathogens. Saudi J. Biol. Sci..

[93] Wang H., Yan A., Liu Z., Yang X., Xu Z., Wang Y., Wang R., Koohi-Moghadam M., Hu L., Xia W. (2019). Deciphering molecular mechanism of silver by integrated omic approaches enables enhancing its antimicrobial efficacy in *E. coli*. PLoS Biol..

[94] Khan S.U., Saleh T.A., Wahab A., Khan M.H.U., Khan D., Khan W.U., Rahim A., Kamal S., Khan F.U., Fahad S. (2018). Nanosilver: New ageless and versatile biomedical therapeutic scaffold. Int. J. Nanomed..

[95] Siddiqi K.S., Husen A., Rao R.A. (2018). A review on biosynthesis of silver nanoparticles and their biocidal properties. J. Nanobiotechnology.

[96] Kambale E.K., Nkanga C.I., Mutonkole B.P., Bapolisi A.M., Tassa D.O., Liesse J.M., Krause R.W., Memvanga P.B. (2020). Green synthesis of antimicrobial silver nanoparticles using aqueous leaf extracts from three Congolese plant species (*Brillantaisia patula*, *Crossopteryx febrifuga* and *Senna siamea*). Heliyon.

[97] Al-Zereini W.A. (2017). *Ononis natrix* and *Salvia verbenaca*: Two Jordanian medicinal plants with cytotoxic and antibacterial activities. J. Herbs Spices Med. Plants.

[98] Vijayan R., Joseph S., Mathew B. (2018). Green synthesis, characterization and applications of noble metal nanoparticles using *Myxopyrum serratulum A. W. Hill* leaf extract. BioNanoScience.

[99] Abou El-Nour K.M., Eftaiha A.A., Al-Warthan A., Ammar R.A. (2010). Synthesis and applications of silver nanoparticles. Arab. J. Chem..

[100] Quinteros M.A., Aristizábal V.C., Dalmasso P.R., Paraje M.G., Páez P.L. (2016). Oxidative stress generation of silver nanoparticles in three bacterial genera and its relationship with the antimicrobial activity. Toxicol. In Vitr..

[101] Liao S., Zhang Y., Pan X., Zhu F., Jiang C., Liu Q., Cheng Z., Dai G., Wu G., Wang L. (2019). Antibacterial activity and mechanism of silver nanoparticles against multidrug-resistant *Pseudomonas aeruginosa*. Int. J. Nanomed..

[102] de Araújo Vieira L.F., Lins M.P., Viana I.M., Dos Santos J.E., Smaniotto S., dos Santos Reis M.D. (2017). Metallic nanoparticles reduce the migration of human fibroblasts in vitro. Nanoscale Res. Lett..

[103] Liu X., Lee P.Y., Ho C.M., Lui V.C., Chen Y., Che C.M., Tam P.K., Wong K.K. (2010). Silver nanoparticles mediate differential responses in keratinocytes and fibroblasts during skin wound healing. ChemMedChem.

[104] Gunasekaran T., Nigusse T., Dhanaraju M.D. (2011). Silver nanoparticles as real topical bullets for wound healing. J. Am. Coll. Clin. Wound Spec..

[105] Vijayakumar V., Samal S.K., Mohanty S., Nayak S.K. (2019). Recent advancements in biopolymer and metal nanoparticle-based materials in diabetic wound healing management. Int. J. Biol. Macromol..

[106] Rigo C., Ferroni L., Tocco I., Roman M., Munivrana I., Gardin C., Cairns W.R., Vindigni V., Azzena B., Barbante C. (2013). Active silver nanoparticles for wound healing. Int. J. Mol. Sci..

